# Evaluation of six commercial kits for the serological diagnosis of Mediterranean visceral leishmaniasis

**DOI:** 10.1371/journal.pntd.0008139

**Published:** 2020-03-25

**Authors:** Maude F. Lévêque, Emilie Battery, Pascal Delaunay, Badre Eddine Lmimouni, Karim Aoun, Coralie L’Ollivier, Patrick Bastien, Charles Mary, Christelle Pomares, Judith Fillaux, Laurence Lachaud

**Affiliations:** 1 Département de Parasitologie-Mycologie, Université de Montpellier, Centre Hospitalier Universitaire de Montpellier, Centre National de Référence des Leishmanioses, MIVEGEC, Montpellier, France; 2 Département de Parasitologie-Mycologie, Université de Montpellier, Centre Hospitalier Universitaire de Montpellier, Montpellier, France; 3 Département de Parasitologie-Mycologie Centre Hospitalier Universitaire de Nice, France, INSERM U1065 Centre Méditerranéen de Médecine Moléculaire, Nice, France; 4 Département de parasitologie, Hôpital militaire d'instruction Mohamed V, Faculté de médecine et de pharmacie, Université Mohammed V, Rabat, Maroc; 5 Département d’Épidémiologie et d’Écologie Parasitaire & LR 16-IPT-06 "Parasitologie médicale, Biotechnologie et Biomolécules », Institut Pasteur de Tunis, Université Tunis-El Manar, Tunis, Tunisie; 6 Département de Parasitologie-Mycologie, Aix Marseille Université, IRD, AP-HM, SSA, VITROME, IHU-Méditerranée Infection, Marseille, France; 7 Département de Parasitologie-Mycologie, Centre Hospitalier Universitaire de Toulouse-Purpan, Pharmaco-chimie et Biologie Pour le Développement (PHARMA-DEV), IRD UMR 152 Université Paul Sabatier, Toulouse, France; Instituto Goncalo Moniz-FIOCRUZ, BRAZIL

## Abstract

**Background:**

Zoonotic visceral leishmaniasis (VL) is endemic in the Mediterranean basin. However, large-scale comparative analyses of the commercial kits for the serological diagnosis of this neglected disease are lacking. This study compared the performances of four enzyme-linked immunosorbent assays (ELISA) and two immunochromatographic tests (ICT) as screening tests for the serodiagnosis of human VL in the Mediterranean region.

**Methodology/Principal findings:**

Serum samples from 319 patients living in France, Tunisia or Morocco were tested using two ICT (IT LEISH and TruQuick LEISH IgG/IgM Meridian) and four ELISA reagents (NovaLisa *Leishmania infantum* IgG, Bordier *Leishmania infantum*, Ridascreen *Leishmania IgG*, and Vircell *Leishmania*). The population with proven VL (n = 181) included 65 immunocompromised patients. Significantly higher percentages of false-negative results were obtained with all assays in immunocompromised patients, compared with the immunocompetent population. In the whole population, sensitivity and specificity ranged from 80.7% to 93.9% and from 95.7% to 100%, respectively. The maximum accuracy was observed with the Bordier and Vircell ELISA kits (96.2%), and the lowest accuracy with Ridascreen reagent (88.7%). New thresholds of positivity are proposed for the Bordier, Vircell and NovaLisa ELISA kits to achieve 95% sensitivity with the highest possible specificity. Western blot (WB), used as a confirmation method, showed 100% sensitivity and identified 10.1% of asymptomatic carriers among the control population from the South of France.

**Conclusions/Significance:**

This is the first study that compared commercially available kits for VL serodiagnosis in the endemic region of the Mediterranean basin. It provides specific information about the tests’ performance to help clinicians and biologists to select the right assay for VL screening.

## Author summary

Human visceral leishmaniasis (VL) is a neglected disease endemic in the Mediterranean region, caused by *Leishmania infantum*. Accurate VL diagnosis is critical for rapid and correct patient management. Various serological tests are used, including commercial kits that require qualifications with specific information about their performance. In this study, we compared the performance of six commercial kits, two immunochromatographic tests (ICT; IT LEISH and TruQuick LEISH IgG/IgM Meridian) and four enzyme-linked immunosorbent assays (ELISA; NovaLisa *Leishmania infantum* IgG, Bordier *Leishmania infantum*, Ridascreen *Leishmania IgG*, and Vircell *Leishmania*), available in France and used as screening tests. The Bordier and Vircell ELISA reagents displayed the highest analytic performance compared with all the other kits. However, no test displayed 95% sensitivity at the thresholds recommended by the manufacturers. To reach this sensitivity value with the highest possible specificity, new thresholds are proposed for the Bordier, Vircell and NovaLisa ELISA reagents. Positive and negative results obtained with the six screening tests were confirmed by Western blot (100% sensitivity). Overall, this study presents a comparative analysis of commercially available kits for the serodiagnosis of human VL and provides additional information on the prevalence of asymptomatic carriers in the Mediterranean basin.

## Introduction

Human visceral leishmaniasis (VL) caused by *Leishmania infantum* is a neglected, yet severe, zoonotic disease that occurs mainly in Latin America and in the Mediterranean region [1]. Dogs are the main reservoir of *L*. *infantum* parasites that are transmitted to humans by the bite of infected phlebotomine sandflies. In humans, the infection can be asymptomatic or lead to an acute form characterized by irregular fever, weight loss, splenomegaly, hepatomegaly, anaemia or pancytopenia [2]. If left untreated, the infection is often fatal. Accurate diagnosis, which is crucial to control the infection, relies on epidemiological data, clinical features, and laboratory tests. Parasite detection by microscopic examination of bone marrow aspirates is the gold standard method, but lacks sensitivity and requires invasive procedures [3]. The culture of parasites isolated from patients might improve the diagnostic sensitivity. The search of specific antibodies in serum or plasma, while requiring non-invasive sampling, remains the most widely used method for VL diagnosis. However, serological tests tend to be less sensitive in children younger than three years [4,5] and in immunocompromised patients [6,7]. Currently, molecular detection is considered the most reliable method [8,9], with a sensitivity higher than 95% for PCR analysis on peripheral blood [10]. Yet, such technique is restricted to well-equipped laboratories and is often combined with serological tests in the routine practices [3].

Various methods are used for VL serodiagnosis. The qualitative detection of *Leishmania* antibodies is based on Western blot (WB) method and immunochromatographic tests (ICT), whereas quantitative results are obtained by indirect immunofluorescence, direct agglutination, and enzyme-linked immunosorbent assays (ELISA). The WB technique, using *L*. *infantum* antigenic fractions, is a highly sensitive and specific method to confirm VL serodiagnosis [11,12]. This technique is time-consuming and quite expensive, thus complex to implement for field surveys and initial serological screenings. Conversely, ICT which are based on the recombinant *Leishmania* rk39 antigen, are well adapted for field testing. Indeed, they are fast and easy to perform, and display good performances for early VL diagnosis [13]. Among the quantitative methods, ELISA based on crude soluble antigens or the recombinant rk39 protein, are widely used for initial screening of VL [3,7], but their performances are influenced by the nature, purity and stability of the used antigens [14]. In this context, commercial kits may offer more uniform procedures for antigen preparation, compared with home-made assays, and provide standardized experimental conditions. However, large-scale studies comparing the performances of commercial assays are lacking [5,7]. A recent study in Brazil compared, for the first time, different kits tested in the same condition and in the same population [5]. Similarly, in the present study, we evaluated the performances of six commercial tests for the diagnosis of human VL in the Mediterranean region. We compared the sensitivity and specificity of two ICT and four ELISA kits, re-evaluated their positivity thresholds, and analysed their advantages and drawbacks for routine diagnosis. Finally, we use a Western blot technique to confirm the serological status of VL and healthy patients, and discuss discrepant positive results obtained in some asymptomatic patients living in the South of France, a VL endemic area in the Mediterranean basin.

## Material and methods

This study complies with the updated Standards for Reporting of Diagnostic Accuracy (STARD) statement [15]. The STARD checklist and a diagram reporting the flow of participant through the study are presented in S1 Checklist and S1 Fig, respectively.

### Study site

This study was carried out at the Parasitology and Mycology Department of Montpellier University Hospital, France, on behalf the French National Reference Centre of Leishmaniasis (Centre National de Référence des Leishmanioses, CNRL) from January to August 2019.

### Study design and human samples

Six commercial kits were evaluated using human serum samples collected between 1998 and 2018 and stored at -20°C at the University Hospitals of Montpellier (n = 171), Nice (n = 138) and Marseille (n = 5), France, at the Mohamed V Military Hospital of Rabat (n = 16), Morocco, and at the Pasteur Institute of Tunis (n = 10), Tunisia. In total, 340 samples were analysed for this comparative study. All samples were sent to the CNRL for epidemiological and analytical purposes and stored at -20°C. Among the serum samples, 202 VL-positive samples were selected to reach a sensitivity of 95% and a precision of 3%. Positive samples were from patients who lived in the Mediterranean basin and displayed at least one clinical sign of VL (*i*.*e*., splenomegaly, hepatomegaly, anaemia leukopenia, or thrombocytopenia). VL diagnosis in these patients was confirmed by microscopic detection of the parasites, culture, or PCR analysis. To reach a minimum specificity of 90% and a precision of 5%, 138 negative serum samples were included. Negative samples were from immunocompetent patients who lived in the South of France, and did not have any typical symptom or previous history of VL.

### Ethics statement

This study was approved by the Committee of Research Ethics of the Montpellier University Hospital (IRB protocol number: 2019_IRB_MTP_03–03). Human serum samples were anonymised, and diagnostic tests were performed in blinded conditions. This work was carried out in accordance with the relevant guidelines and regulations, and does not provide any information that may allow the identification of the enrolled patients.

### Serological assays

Commercial kits available in France to detect human antibodies against *Leishmania* were used in this study. Four ELISA kits were included: NovaLisa *Leishmania infantum* IgG, Bordier *Leishmania infantum*, Ridascreen *Leishmania IgG*, and Vircell *Leishmania*. Sera were incubated in microplates, containing up to 96 wells coated with kits’ specific *Leishmania* antigens, for 1h using Novalisa, 45min for Vircell, 30min for Bordier and 15 min for Ridascreen. Then, wells were washed and incubated with the conjugate for 30 min using Novalisa, Bordier and Vircell, and 15 min for Ridascreen. After a second washing step, plates were incubated with the substrate for 15 min using Novalisa and Ridascreen, 20 min for Vircell and 30min for Bordier. All four assays were carried out on the Evolis (BIO-RAD) automate and the absorbance was measured at 450 nm. Results were calculated as the sample absorbance value divided by the mean of the absorbance values of the cut-off controls, multiplied by ten for Novalisa, Vircell and Ridascreen. For the screening by ICT, samples were processed manually using the IT LEISH and TruQuick LEISH IgG/IgM Meridian tests, according to the manufacturers’ instructions. For western blotting, the LDBio *Leishmania* IgG kit was used with the Autoblot 3000 (MedTEC) apparatus according to the manufacturer’s instructions. Specific bands at 14 kDa or 16 kDa demonstrated the presence of anti-*Leishmania* antibodies in the sample. The characteristics of each test are listed in Table 1.

**Table 1:** Characteristics of the commercial kits for the serological diagnosis of human visceral leishmaniasis. The asterisk corresponds to the total duration for the analysis of 96 wells.

Commercial kit	Manufacturer	Method	Threshold	Sample type and volume	Time	Antigen
**NOVALISA**® *Leishmania infantum* IgG	Novatec Immundiagnostica GMBH	ELISA	Negative: <9 Equivocal: [9–11[ Positive: ≥11	Serum 10μL	3.5h*	*L*. *infantum* antigen
**BORDIER**® *Leishmania infantum*	Bordier Affinity products SA	ELISA	Negative: <1 Positive: ≥1	Serum 5μL	3.5h*	Soluble antigens from *L*. *infantum* promastigotes
**RIDASCREEN**® *Leishmania* IgG	R-Biopharm AG	ELISA	Negative: <9 Equivocal: [9–11[ Positive: ≥11	Serum 10μL	2.5h*	Recombinant *L*. *infantum* antigen
**VIRCELL**® *Leishmania* IgG/IgM	Vircell S.L.	ELISA	Negative: <9 Equivocal: [9–11[ Positive: ≥11	Serum 5μL	2.5h*	*L*. *infantum* antigen
**IT LEISH®**	BIO-RAD Laboratories, Inc.	ICT	Positive: Control + IgG bands	Serum/Blood 8–12μL	25min	Recombinant K39 antigen
**MERIDIAN**® TruQuick™ LEISH IgG/IgM	Meridian Bioscience	ICT	Positive: Control + IgG and/or IgM bands	Serum/Blood 40μL	15min	Recombinant *L*. *donovani* antigen
***Leishmania* Western blot** IgG	LD BIO Diagnostics	Western Blot	Positive: 14-kD and/or 16-kD band	Serum 25μL	3.5h	Antigens from *L*. *infantum* promastigotes

### Statistical analysis

The characteristics of the studied population were described using percentages and medians along with interquartile ranges instead of means and standard deviations when distributions were found to be non-Gaussian. Screening tests were evaluated against parasitological (*i*.*e*. microscopy, culture) and molecular diagnosis. Sensitivity and specificity were calculated using all the serum samples included in the study. The sensitivity rate was calculated as the number of patients with VL who tested positive divided by the total number of patients with VL (n = 181). The specificity rate was calculated as the number of non-VL patients who tested negative divided by the total number of non-VL patients (n = 138). The accuracy rate was calculated as the number of patients with VL who tested positive plus the number of non-VL patients who tested negative divided by the total number of patients tested (n = 319) [5]. Results were analysed using Receiving Operating Characteristic (ROC) curves to determine the most suitable threshold(s) for each quantitative screening test. Sensitivity and specificity values were compared by testing the equality of proportions. The areas under the curve (AUC) were compared with the χ2 test. The significance threshold was set at 5%. All statistical tests and procedures were performed using the Intercooled Stata 9.2 statistical package (StataCorp, College Station, TX).

## Results

### Characteristics of the population, samples, and assays

We screened serum samples from patients living in France, Tunisia and Morocco. Among the VL population, the female to male sex ratio was 2:3, and age ranged from 1 to 93 years with a median of 33 years [IQR 28–44]. Because of the low quantity of some samples, among the 340 sera included in this study, 339 sera were screened with LDBio, 338 with IT-LEISH, 335 with TruQuick, 339 with Bordier and Ridascreen, 335 with NovaLisa, and 322 with Vircell. Among these sera, a complete dataset was available for 319 samples. The VL population (n = 181) included 98 immunocompetent and 65 immunocompromised patients (HIV: n = 49, transplantation: n = 4, immunosuppressive treatments n = 12), and 18 patients with unknown immune status who were included in the immunocompetent group (n = 116). S2 Fig presents the distribution of the index values obtained using the four ELISA kits, according to patients’ immune status, and shows that kits’ specific values cannot be compared to each other.

### Comparison of the tests’ performances

**Fig 1:**
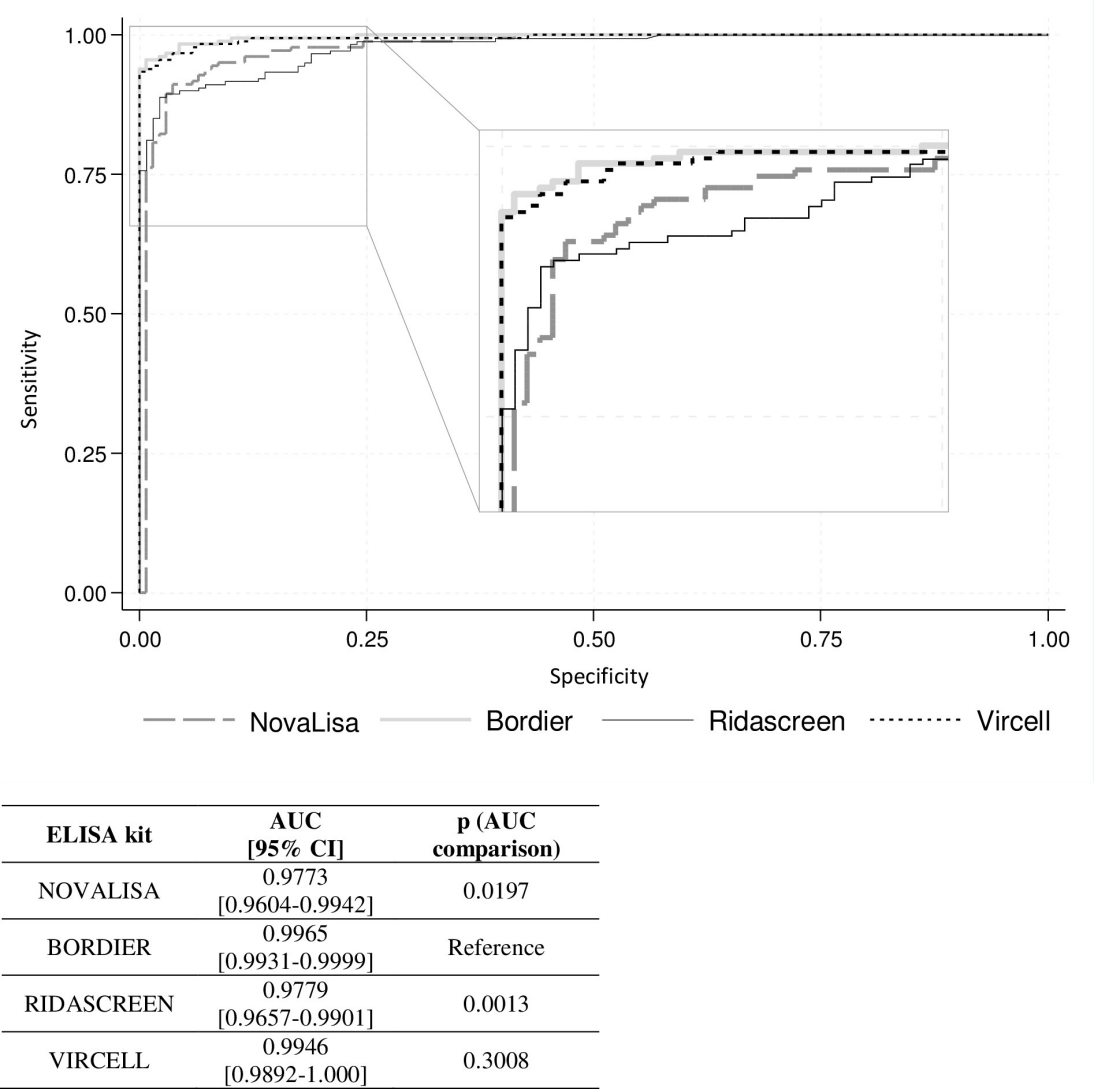
Comparison of the ROC curves for the four ELISA assays.

To compare the performance of the different ICT and ELISA kits, we assessed their sensitivity, specificity and accuracy in the 319 samples tested with all kits (Table 2). We interpreted the results of the four ELISA kits using the cut-off values for antibody detection recommended by the manufacturers (Table 1). The results that were found in the grey zone defined by the manufacturer of Vircell (n = 16), Ridascreen (n = 10) and NovaLisa (n = 11) were considered positive. Overall, the sensitivity and specificity of the ICT and ELISA kits ranged from 80.7% to 93.9% and from 95.7% to 100%, respectively. Among all tests, the highest values were obtained with the Bordier and Vircell kits, both displaying 96.2% of accuracy. When comparing the maximum likelihood ratio of ELISA calculated by the ROC curves, we found that the Bordier kit displayed significantly higher analytical performances than the Ridascreen and NovaLisa assays, whereas it was comparable to Vircell in the whole population (Fig 1). Conversely, the four ELISA kits performed similarly when only samples from immunocompetent patients with VL were considered (S1 Table). In this population, the sensitivity of each test was improved (S2 Table). In immunocompromised patients, the overall distribution of the index values was significantly lower than in the immunocompetent population (S2 Fig). Moreover, in the VL-positive group, the number of false-negative results was significantly higher in immunocompromised patients than in the immunocompetent population (Table 3).

**Table 2:** Sensitivity, specificity and accuracy of the tested commercial kits.

Manufacturer	Sensitivity (%) [95%CI]	Specificity (%) [95%CI)	Accuracy (%) [95%CI]	p (accuracy comparison)
**NOVALISA®**	89.5% [86.1–92.9]	96.4% [94.3–98.4]	92.5% [89.7–95.4]	0.043
**BORDIER®**	93.4% [90.6–96.1]	100% [100–100]	96.2% [94.1–98.3]	Reference
**RIDASCREEN®**	80.7% [76.3–85.0]	99,3% [98.3–100.2]	88.7% [85.2–92.2]	<0.001
**VIRCELL®**	93.9% [91.3–96.5]	99,3% [98.3–100.2]	96.2% [94.1–98.3]	1
**IT LEISH®**	85.1% [81.2–88.9]	99,3% [98.3–100.2]	91.2% [88.1–94.3]	0.009
**TRUQUICK®**	90.1% [86.8–93.3]	95,7% [93.4–97.9]	92.5% [89.7–95.4]	0.043

**Table 3:** Comparison of false-negative results in patients with visceral leishmaniasis according to their immune status.

Manufacturer	Immunocompetent (n = 116)	Immunocompromised (n = 65)	p
**NOVALISA®**	7 (6.0%)	12 (18.5%)	0.009
**BORDIER®**	2 (1.7%)	10 (15.4%)	<0.001
**RIDASCREEN®**	8 (6.9%)	27 (41.5%)	<0.001
**VIRCELL®**	1 (0.9%)	10 (15.4%)	<0.001
**IT LEISH ®**	8 (6.9%)	19 (29.2%)	<0.001
**TRUQUICK®**	6 (5.2%)	12 (18.5%)	0.004

### Threshold adjustment of ELISA reagents

Sensitivity and specificity of 95% and 98%, respectively, are recommended for VL diagnosis [5,16]. However, in the whole population (n = 319), none of the ELISA kits reached 95% sensitivity using the thresholds recommended by the manufacturers, although specificity ranged from 96.38% to 100%, (Table 2). Therefore, we estimated the positivity thresholds to obtain a sensitivity of 95% with the highest possible specificity (Table 4). To reach these parameters, all cut-off values had to be reduced. For instance, for the Bordier kit, a decrease of the positive threshold from 1 to 0.746 gave a specificity of 99.3%. After adjusting the positive cut-off, the specificity of the Ridascreen and NovaLisa assays, were significantly lower than that of Bordier, while there was no statistical difference in between Bordier and Vircell.

**Table 4:** Sensitivity and specificity of the four ELISA assays using the proposed thresholds.

ELISA kits	Manufacturer threshold	Threshold	Sensitivity (%) [95%CI]	Specificity (%) [95%CI]	p (specificity)
Vircell®	9–11	7.686	95.03 [92.6–97.4]	97.83 [96.2–99.4]	0.062
NovaLisa®	9–11	5.604		91.30 [88.2–81.88]	<0.001
Ridascreen®	9–11	1.491		81.88 [77.6–86.1]	<0.001
Bordier®	1	0.746		99.28 [98.3–100]	Reference

### Results confirmation by western blot

**Fig 2:**
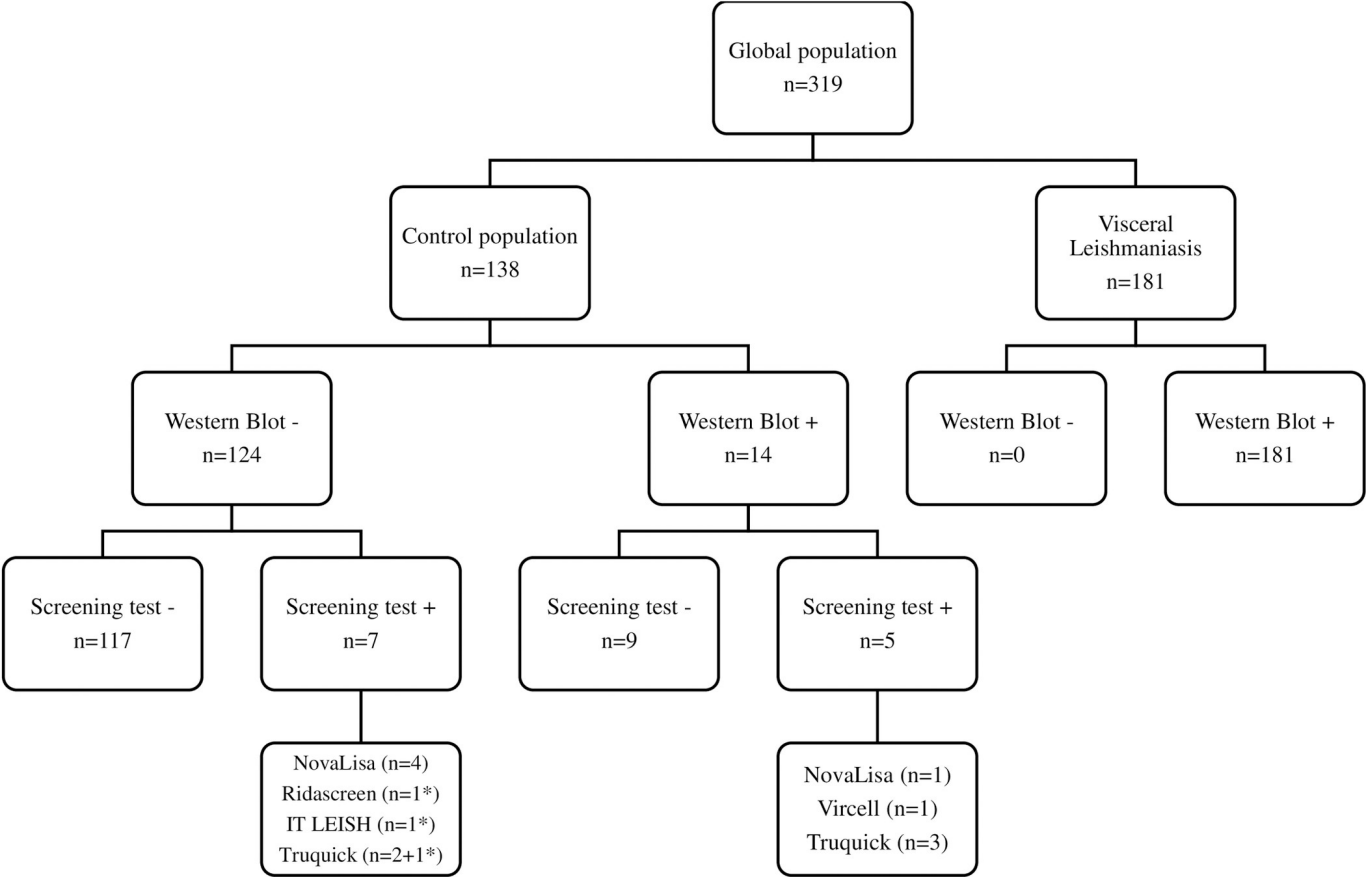
Confirmation of screening tests results by Western blot. The asterisk corresponds to the same patient who was found positive with Ridascreen, IT LEISH and TruQuick kits. Positive ELISA tests’ results were defined according to the cut-off values recommended by the manufacturer.

Western blotting is used in the routine practice to confirm equivocal or positive results obtained by initial screening tests. Western blot analysis of the serum samples from patients with proven VL (n = 181) confirmed the results in 100% of cases (Fig 2). On the other hand, in the control population (n = 138), 12 serum samples were identified as positive by at least one ICT or ELISA kit. Analysis of these samples by western blotting indicated that seven samples were negative (n = 4 positive with NovaLisa, n = 2 positive with TruQuick, and n = 1 positive with Ridascreen, IT LEISH and TruQuick kits). On the other hand, we confirmed positive detection in five samples (n = 1 NovaLisa, n = 1 Vircell, n = 3 TruQuick). Therefore, we analysed all samples in the control group (n = 138) to assess what was the percentage of patients who did not present any symptoms of VL but displayed positive detection by Western blot. In total, we found 14 positive samples (10.1%) in the control population.

## Discussion

During the last 70 years, various methods have been used for the serodiagnosis of VL caused by *L*. *infantum* in humans [17], including commercial kits that require qualifications with specific information about their performance [5]. In this study, we evaluated the analytical performances of commercially available kits based on different methods: ELISA, ICT and WB. One of the limitations of this study is that we did not include immunocompromised patients in the healthy population. Consequently, we could not compare the tests’ accuracy in function of the immune status. In addition, the control group did not include patients with diseases known to cross-react with anti-*Leishmania* antibodies (*i*.*e*. tuberculosis, trypanosomiasis, toxoplasmosis, leprosy or auto-immune diseases [3,7]). The use of a healthy population may have influenced the high specificity observed for all tests (>95%).

Among the six assays used for antibody screening, none reached the recommended sensitivity of 95% for VL diagnosis (14). Therefore, we lowered the positivity thresholds of the ELISA reagents to obtain a sensitivity of 95% with the highest possible specificity. As no grey zone is defined by the manufacturer of Bordier, we recommend interpreting the index values between 0.7 and 1 as equivocal or positive results. By using these cut-off values, the Bordier kit displayed the highest specificity (>99%). We propose to broaden the cut-off values recommended by the Vircell manufacturer from 9–11 to 8–11, yielding a specificity comparable to that of Bordier. We also suggest reducing the NovaLisa positivity threshold from 9 to 6, although this would entail a significant decrease of specificity and the use of additional confirmation tests, compared with the Bordier and Vircell assays. Moreover, the cut-off values (1.5–11) of the Ridascreen kit required to achieve a sensitivity of 95% significantly reduced specificity (81.9%), therefore making this test less suitable for routine screening.

Few studies evaluated ICT performance for the diagnosis of zoonotic VL [16]. However, commercial ICT kits represent the best choice for rapid, easy and efficient serological screening of VL [17]. In this study, the performances of TruQuick and IT-LEISH were lower than those of the Bordier and Vircell ELISA kits, but their estimated accuracy was comparable to that of NovaLisa and higher than that of the Ridascreen ELISA kits in the whole population. ICT present several advantages compared with ELISA kits in term of practicability. First, TruQuick and IT-LEISH can be performed also on whole blood, thus facilitating screening in field settings. It should be noted that the performances of several commercial ICT kits on whole blood were found comparable to those obtained in serum samples [18]. Second, they provide qualitative results in 15–25 minutes compared to the 2.5–3.5 hours with ELISA kits. Moreover, the sample volume required for ICT is lower than for ELISA when using automated processors.

Recently, Freire *et al*. compared one ICT (IT LEISH) and three ELISA (Vircell, Novalisa and Ridascreen) kits in Brazil [5]. The overall specificity of these commercial kits (>95% in the whole population) was comparable in our study and in the work by Freire *et al*., except for Ridascreen (specificity <85% in Brazil). Conversely, while Ridascreen displayed the highest sensitivity value in Brazil, we found that this test had the lowest sensitivity among all kits we evaluated. On the other hand, the sensitivity and specificity of the IT LEISH ICT were similar in both studies, although our values were slightly higher in the whole population. These differences might be explained by antigen variability in parasites from different geographic regions and by cross-reactivity with other endemic pathogens, as highlighted by the World Health Organization (WHO) evaluation of ICT [19]. Finally, like Freire *et al*. [5] and other works [6,7] that reported lower performances of serodiagnostic tests in immunocompromised patients, we found a higher number of false-negative results in immunocompromised individuals with VL. In Europe, a meta-analysis concluded that serological tests should not be used to rule out a diagnosis of VL among HIV-infected patients [20]. In a recent study in Spain, the sensitivity of the rk39-ICT dropped from 78% to 67.3% in this population [21]. These results support the use of molecular assays as an alternative method for VL diagnosis in immunocompromised patients.

Regarding WB, this technique is currently used to confirm the positive results obtained by serological screening. Its specificity should be discussed by taking into account the existence of asymptomatic carriers. The Leishmanin skin test [22], blood culture [23], and WB [11,12] could indeed detect infection or parasite contact in patient without VL. Here, 10.1% of the control population was positive by WB. This value is consistent with a larger study performed in Italy showing 7.41% of asymptomatic carriers by WB [24]. Thus, a positive result by WB should be discussed and interpreted on the basis of the biological and clinical data to discriminate between disease and asymptomatic carriage.

In conclusion, the Vircell and Bordier ELISA kits displayed significantly higher accuracy rates in the whole population than the other tests. Nevertheless, we had to decrease the cut-off values to improve the performance of the four ELISA tests. For field studies, the TruQuick ICT is the most suitable with satisfactory performances. In immunocompromised patients, the index values of all ELISA reagents were significantly lower than in the immunocompetent population and false-negative results were more frequent, as shown previously [20]. This kits comparison might help to select the most suitable method, using the most appropriate thresholds, and to better understand the limitations of each test.

## Supporting information

S1 Checklist STARD checklist for reporting of studies of diagnostic accuracy. The STARD checklist describes the design of the current study in order to improve reporting accuracy and completeness.
(DOCX)

S1 Fig Diagram reporting the flow of participant through the study. P: positive; N: negative; I: indeterminate; Orange case: target condition present; Green case: target condition absent.
(TIF)

S2 Fig Distribution of anti-*Leishmania infantum* antibody titres for the four ELISA assays according to patients’ immune status. (TIF)

S1 Table Comparison of the ROC curves for the four ELISA assays in the immunocompetent population. (DOCX)

S2 Table Sensitivity, specificity and accuracy of the tested commercial kits in the immunocompetent population. (DOCX)

S3 Table Spreadsheet of raw data. Negative = 0; Positive = 1; Grey cell = unknown; Pink cell = immunosuppression; Orange cell = grey zone; Yellow cell = discrepancy.
(XLSX)
