# Supplementary figures and images for: Evaluation of six commercial kits for the serological diagnosis of Mediterranean visceral leishmaniasis
Source: PLoS Negl Trop Dis. 2020 Mar 25;14(3):e0008139. doi: 10.1371/journal.pntd.0008139 (PMC7135331; doi:10.1371/journal.pntd.0008139)

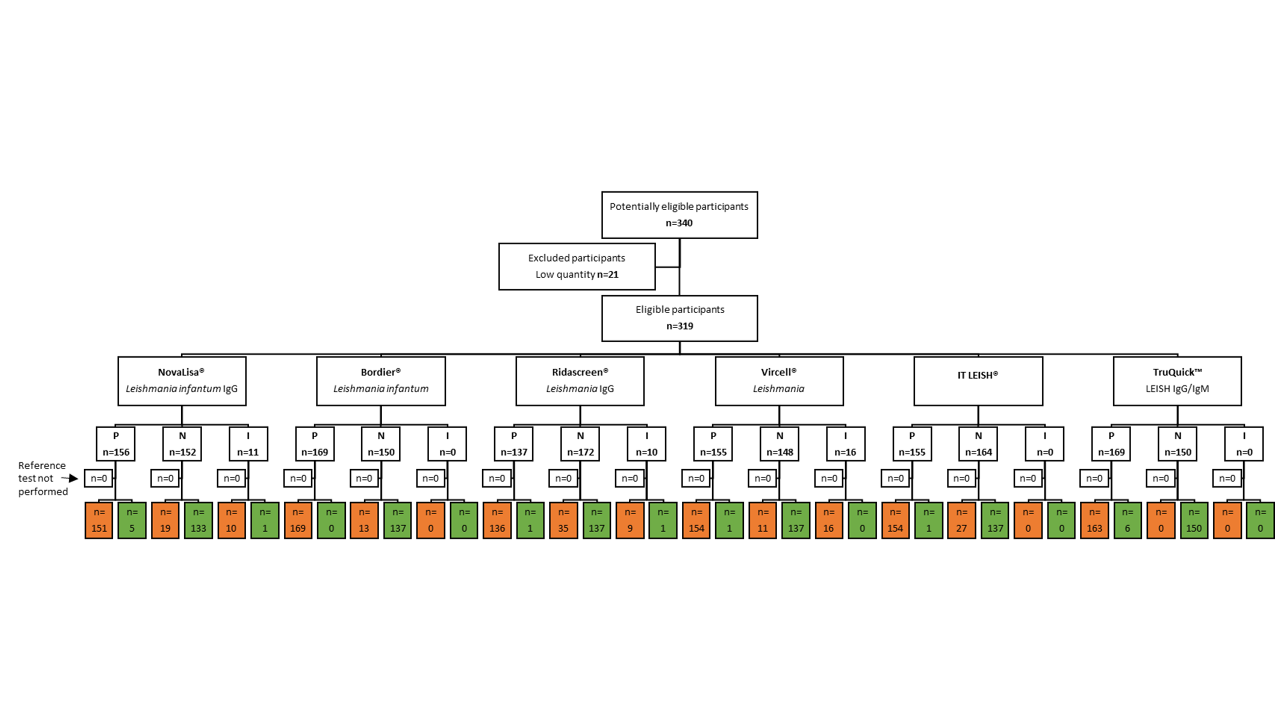

Supplement: S1 Fig — P: positive; N: negative; I: indeterminate; Orange case: target condition present; Green case: target condition absent. (TIF) [file pntd.0008139.s002.tif]

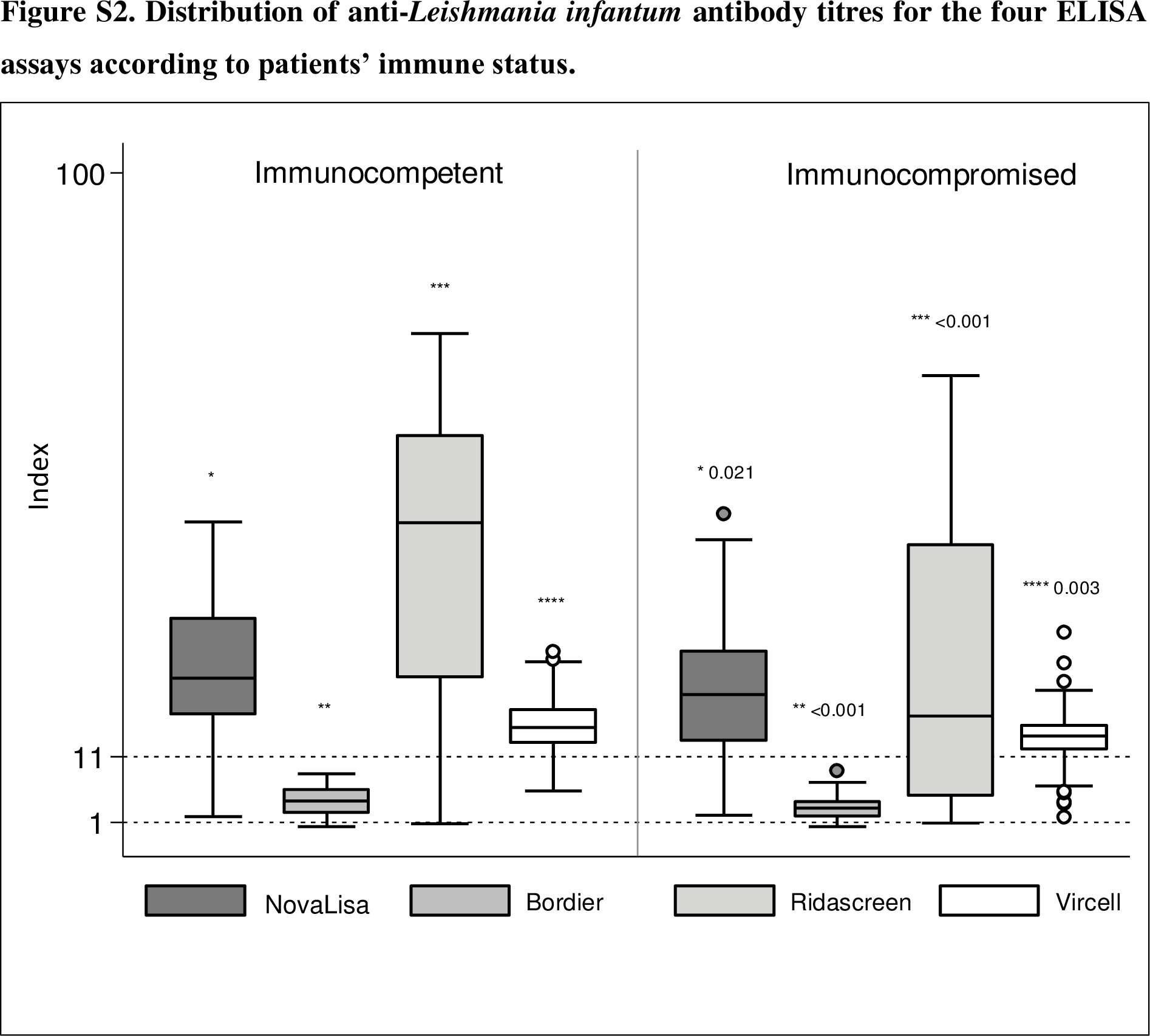

Supplement: S2 Fig — (TIF) [file pntd.0008139.s003.tif]
