# Supplementary material for: Evaluation of six commercial kits for the serological diagnosis of Mediterranean visceral leishmaniasis
Source: PLoS Negl Trop Dis. 2020 Mar 25;14(3):e0008139. doi: 10.1371/journal.pntd.0008139 (PMC7135331; doi:10.1371/journal.pntd.0008139)
Supplement: S1 Table — (DOCX) [file pntd.0008139.s004.docx]

## Table S1. Comparison of the ROC curves for the four ELISA assays in the immunocompetent population.

| **ELISA kit** | **AUC**  **[95% CI]** | **p (AUC comparison)** |
| --- | --- | --- |
| NOVALISA | 0.9844  [0.9680-1.000] | 0.0666 |
| BORDIER | 0.9991  [0.9974-1.000] | Reference |
| RIDASCREEN | 0.9909  [0.9803-1.000] | 0.1311 |
| VIRCELL | 0.9995  [0.9985-1.000] | 0.4304 |
