# Supplementary material for: Evaluation of six commercial kits for the serological diagnosis of Mediterranean visceral leishmaniasis
Source: PLoS Negl Trop Dis. 2020 Mar 25;14(3):e0008139. doi: 10.1371/journal.pntd.0008139 (PMC7135331; doi:10.1371/journal.pntd.0008139)
Supplement: S2 Table — (DOCX) [file pntd.0008139.s005.docx]

## Table S2. Sensitivity, specificity and accuracy of the tested commercial kits in the immunocompetent population.

| **Manufacturer** | **Sensitivity (%)**  [95%CI] | **Specificity (%)**  [95%CI) | **Accuracy (%)**  [95%CI] | **p**  **(accuracy comparison)** |
| --- | --- | --- | --- | --- |
| **NOVALISA** | 94.0% [91.0-96.9] | 96.4% [94.1-98.7] | 92.3% [89.0-95.6] | <0.001 |
| **BORDIER** | 98.3% [96.7-99.9] | 100% [100-100] | 99.2% [98.1-100] | Reference |
| **RIDASCREEN** | 93.1% [90.0-96.2] | 99,3% [98.2-100] | 96.5% [94.2-98.8] | 0.0359 |
| **VIRCELL** | 99.1% [98.0-100] | 99,3% [98.2-100] | 99.2% [98.1-100] | 1 |
| **IT LEISH** | 93.1% [90.0-96.2] | 99,3% [98.2-100] | 96.5% [94.2-98.8] | 0.0359 |
| **TRUQUICK** | 94.8% [92.1-97.6] | 95,7% [93.1-98.2] | 92.3% [89.0-95.6] | <0.001 |
